# Beta-sheet assembly of Tau and neurodegeneration in *Drosophila melanogaster*

**DOI:** 10.1016/j.neurobiolaging.2018.07.022

**Published:** 2018-12

**Authors:** Daniela Passarella, Michel Goedert

**Affiliations:** MRC Laboratory of Molecular Biology, Cambridge, UK

**Keywords:** Tau, Beta-sheet assembly, Neurodegeneration, *Drosophila*

## Abstract

The assembly of Tau into abundant β-sheet-rich filaments characterizes human tauopathies. A pathological pathway leading from monomeric to filamentous Tau is believed to be at the heart of these diseases. However, in *Drosophila* models of Tauopathy, neurodegeneration has been observed in the absence of abundant Tau filaments. Here we investigated the role of Tau assembly into β-sheets by expressing wild-type and Δ306–311 human Tau-383 in the retina and brain of *Drosophila*. We analyzed both lines for eye abnormalities, brain vacuolization, Tau phosphorylation and assembly, as well as climbing activity and survival. Flies expressing wild-type Tau-383 showed MC-1 staining, Tau hyperphosphorylation, and neurodegeneration. By contrast, flies expressing Δ306–311 Tau-383 had less MC-1 staining, reduced Tau hyperphosphorylation, and no detectable neurodegeneration. Their climbing ability and lifespan were similar to those of nontransgenic flies. Fluorescence spectroscopy after addition of Thioflavin T, a dye that interacts with β-sheets, showed no signal when Δ306–311 Tau-383 was incubated with heparin. These findings demonstrate that the assembly of Tau into β-sheets is necessary for neurodegeneration.

## 1 Introduction

Tauopathies are human neurodegenerative diseases that are characterized by the ordered assembly of Tau protein into abundant filamentous deposits with the cross-β architecture of amyloid in either nerve cells or in both nerve cells and glial cells (Goedert et al., 2017). Dominantly inherited mutations in *MAPT*, which encodes Tau protein, cause frontotemporal dementia and Parkinsonism linked to chromosome 17, showing that dysfunction of Tau is sufficient to cause neurodegeneration and dementia. Six Tau isoforms are expressed in adult human brain. They range from 352 to 441 amino acids and are produced by alternative mRNA splicing of transcripts from *MAPT* (Goedert et al., 1989). The 6 isoforms are natively unfolded and differ by the presence or absence of inserts of 29 or 58 amino acids in the amino-terminal half (1N and 2N), and the inclusion or not, of the 31 amino acid repeat encoded by exon 10, in the carboxy-terminal half. Inclusion of exon 10 results in the production of 3 Tau isoforms with 4 repeats each (4R), and its exclusion in a further 3 isoforms with 3 repeats each (3R). The 4R Tau isoforms have R1, R2, R3, and R4, whereas 3R Tau isoforms have R1, R3, and R4.

Although the Tau species responsible for neurodegeneration are unknown, short filaments constitute the major species of seed-competent Tau in the brains of mice transgenic for human P301S Tau (Jackson et al., 2016). In human Tau-overexpressing *Drosophila*, nerve cell loss, behavioral deficits, and reduced lifespan have been reported in the absence of Tau filaments, suggesting that the events leading from Tau expression to neurodegeneration may not involve filament formation (Ali et al., 2012, Shulman and Feany, 2003, Wittmann et al., 2001). One study showed that on co-expression of the *Drosophila* homolog of glycogen synthase kinase-3, filamentous Tau formed (Jackson et al., 2002). Tau oligomers have been detected in the brains of individuals with Alzheimer's disease (AD) and progressive supranuclear palsy, consistent with the view that nonfilamentous Tau aggregates may contribute to neurotoxicity (Castillo-Carranza et al., 2017). However, the relevance of Tau assembly into β-sheets for neurodegeneration is unknown.

A hexapeptide motif at the beginning of R3, ^306^VQIVYK^311^, is essential for the assembly of Tau (Li and Lee, 2006, von Bergen et al., 2000). In its absence, recombinant Tau is unable to undergo assembly into β-sheet-rich structures. Thus, full-length recombinant 0N3R Tau and the 4R K18 fragment (residues 244–372) that lacked this hexapeptide were unable to assemble into filaments in the presence of heparin. Conversely, the hexapeptide from R3 formed amyloid fibrils and its structure was determined by X-ray crystallography (Sawaya et al., 2007). In the cryogenic electron microscopy structures of Tau filaments from AD and Pick's disease brains, the β-sheet comprising ^306^VQIVYK^311^ is located in the protofilament core (Falcon et al., 2018, Fitzpatrick et al., 2017). This hexapeptide is also involved in the binding of Tau to microtubules, but it is in a different conformation from that found in Tau filaments (Kadavath et al., 2015). The related hexapeptide motif ^275^VQIINK^280^ is found at the N-terminus of R2 (von Bergen et al., 2001). In the structure of Tau filaments from AD brain, it is located in the fuzzy coat (Fitzpatrick et al., 2017). Because the cores of Tau filaments from Pick's disease brain only comprise 3R Tau, they lack R2 (Falcon et al., 2018). Deletion of either hexapeptide motif reduces Tau assembly, but only ^306^VQIVYK^311^ is necessary for filament formation (Ganguly et al., 2015, Li and Lee, 2006). Deletion of ^275^VQIINK^280^ and ^306^VQIVYK^311^ abolished the seeding activity of recombinant full-length Tau, showing that its assembly into β-sheets is necessary for seeding (Falcon et al., 2015).

Here we expressed human Tau-383 (isoform 0N4R) with or without ^306^VQIVYK^311^ in photoreceptors and nerve cells of the brain of *Drosophila*. The expression of human Tau took place in the presence of the *Drosophila* Tau/MAP2/MAP4 homolog (Heidary and Fortini, 2001). We observed neurodegeneration following the expression of wild-type Tau-383. By contrast, expression of Δ306–311 Tau-383 did not result in neurodegeneration, indicating that assembly of Tau into β-sheets is necessary for neurodegeneration.

## 2 Materials and methods

### 2.1 Transgenic fly lines

Human Tau-383 cDNAs (wild-type and Δ306–311 0N4R) were subcloned into pUAST and the plasmid DNAs microinjected into yw fly embryos. The UAS-Tau variants were integrated at the 68A locus of the third chromosome (BestGene Inc, Chino Hills, CA, USA) using ϕC31-mediated transgenesis (Groth et al., 2004). For human Tau expression, the UAS/GAL4 system was used. Driver lines were ELAV-Gal4^C155^ for pan-neuronal expression and GMR-Gal4 for retinal expression. Fly stocks were maintained and crosses, as well as experiments, carried out in standard sugar-yeast medium at 25 °C on a 12:12 hours light-dark cycle at constant humidity.

### 2.2 Tissue extraction, western blotting, and immunohistochemistry

Fly heads (2 μL/per head, 30 heads/blot) were homogenized in cold Tris buffer (25 mM Tris-HCl, pH 7.4, 15 mM NaCl, 1 mM EGTA, 1 mM ethylenediaminetetraacetic acid, supplemented with protease and phosphatase inhibitors) and centrifuged at 3000 × g for 3 minutes, followed by a 1 hour spin of the supernatant at 100,000 × g. Sarkosyl extraction, SDS-PAGE, and western blotting were carried out as described (Colodner and Feany, 2010). The primary anti-Tau antibodies were HT7 (1:500), Tau46 (1:500), AT270 (1:5000), AT8 (1:100), and AT180 (1:500). HT7 and Tau46 are phosphorylation-independent and specific for the amino- and carboxy-terminal regions of Tau, respectively. AT270, AT8, and AT180 are phosphorylation-dependent and recognize pT181 (AT270), pS202, pT205, and pS208 (AT8) (Malia et al., 2016), and pT231 (AT180) in Tau. The anti-beta actin antibody was used at 1:1000. Except for anti-beta actin (Abcam), all antibodies were from Thermo Scientific Pierce. For immunohistochemistry, paraffin-embedded brain sections (4 μm) were incubated with primary antibody (HT7, 1:400; AT8, 1:400, MC-1, 1:100) for 24 h at 4 °C, washed, incubated with secondary antibody, and the signal visualized using a Vector VIP substrate kit (Vector Laboratories) with an Olympus BX41 microscope equipped with an integrated 3.0 megapixel CMOS camera.

### 2.3 Imaging fly eyes

Transgenic (GMR-Gal4;hTau^WT^ and GMR-Gal4;hTau^Δ(306−311)^) and control (GMR-Gal4) flies from at least 3 independent crosses (30 flies per group, per cross) were processed for scanning electron microscopy (SEM) and light microscopy, respectively. For SEM, flies were fixed in ethanol, point-dried, attached to aluminum stubs, coated with gold in a sputter-coating apparatus and imaged using INCAPenta FET-×3 SEM microscope (magnification: 110× and 225×). For light microscopy, the eyes of anesthetized flies were analyzed using an Olympus BX41 microscope equipped with an integrated 3.0 megapixel CMOS camera (magnification: 100×).

### 2.4 Negative geotaxis

For each gender, 30-day-old transgenic (ELAV-Gal4;hTau^WT^, ELAV-Gal4;hTau^Δ(306−311^) and control (ELAV-Gal4) flies were separated into groups of 10 and tested, as described (Ali et al., 2011). In brief, the flies were gently tapped to the bottom of the vial and those that climbed above the 4 cm mark by 30 seconds and the 8 cm mark by 60 seconds scored. Climbing performance was recorded on 3 biological cohorts and repeated 10 times for each group with a 1 minute break between each trial. The mean (average climbing pass rate) was plotted as a single data point.

### 2.5 Lifespan

The ELAV-Gal4 lines were used. Thirty freshly hatched flies (15 of each gender) were collected and kept at 25 °C. Food was changed daily and the number of dead flies recorded. Survival was recorded using 3 biological cohorts and differences between groups recorded using the long-rank Mantel Cox test. Results were expressed as the proportion of survivors.

### 2.6 Recombinant Tau production and assembly

0N4R human Tau (383 amino acid isoform) and its Δ306–311 mutant were expressed and purified, essentially as described (Bugiani et al., 1999). Protein concentrations were determined using a NanoDrop spectrophotometer. Purified Tau (3 mg/mL) was incubated with heparin (200 μg/mL, BDH) and β-sheet assembly was monitored in a plate reader using Thioflavin T fluorescence for 5 days at 37 °C.

## 3 Results

### 3.1 Transgenic lines

**Fig. 1:**
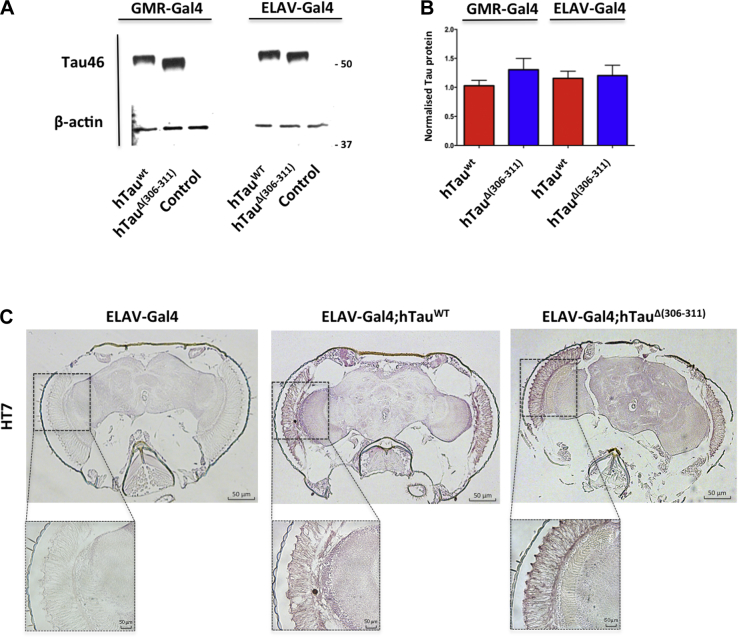
Expression of human Tau in *Drosophila*. (A) Western blot for hTau (antibody T46) of the heads of 30-day-old flies. Beta-actin served as the control. (B) Wild-type Tau-383 and Δ(306–311) Tau-383 flies expressed similar levels of transgenic human protein (n = 3, different crosses). (C) Immunohistochemistry with anti-Tau antibody HT7 of brain using 4 μm frontal paraffin sections of 30-day-old flies. Wild-type (ELAV-Gal4) and transgenic (ELAV-Gal4;hTau^WT^ and ELAV-Gal4;hTAU^Δ(306−311)^ flies were used.

We generated 2 transgenic lines: one expressing wild-type and one expressing Δ306–311 human Tau (383 amino acid isoform). To avoid positional effects, we used ϕC31 integrase–mediated transgenesis (Groth et al., 2004). Driver lines were GMR-Gal4 and ELAV-Gal4. The heads of 2-day-old flies were analyzed by western blotting and immunohistochemistry. Western blotting with antibody Tau46 showed similar levels of Tau expression in both lines (Fig. 1A). Immunohistochemistry using HT7 confirmed Tau expression in retina and central nervous system (Fig. 1B).

### 3.2 Deletion of residues 306–311 in Tau-383 flies reduces MC-1 immunoreactivity and Tau phosphorylation

**Fig. 2:**
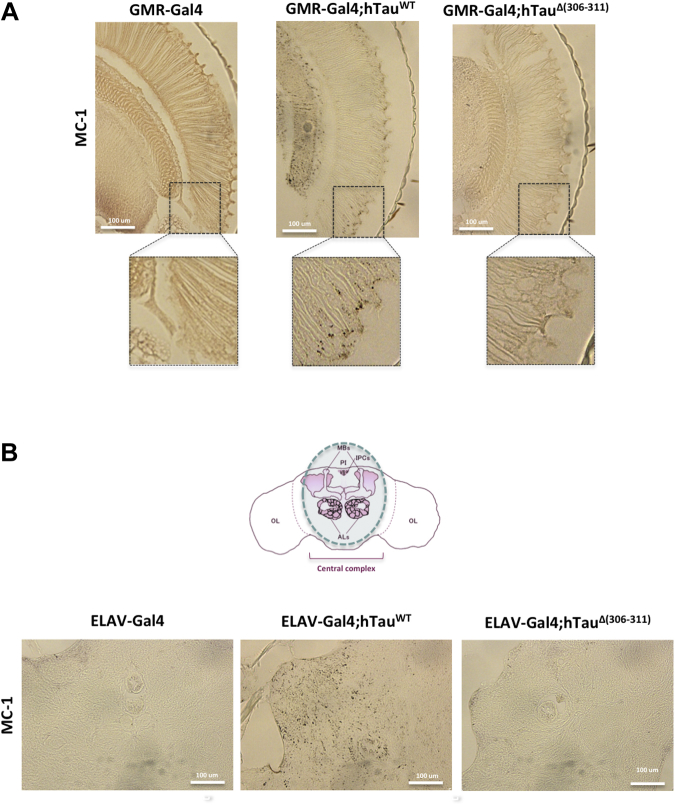
Δ(306–311) in Tau-383 flies reduces MC-1 staining. Immunohistochemistry with MC-1 of retina (A) and brain (B) of 30-day-old flies. Wild-type (GMR-Gal4 and ELAV-Gal4) and transgenic (GMR-Gal4;hTau^WT^, GMR-Gal4;hTau^Δ(306−311)^, ELAV-Gal4;hTau^WT^, and ELAV-Gal4;hTau^Δ(306−311)^) flies were used (4 μm paraffin sections). Representative images are shown. Scale bars, 100 μm.

**Fig. 3:**
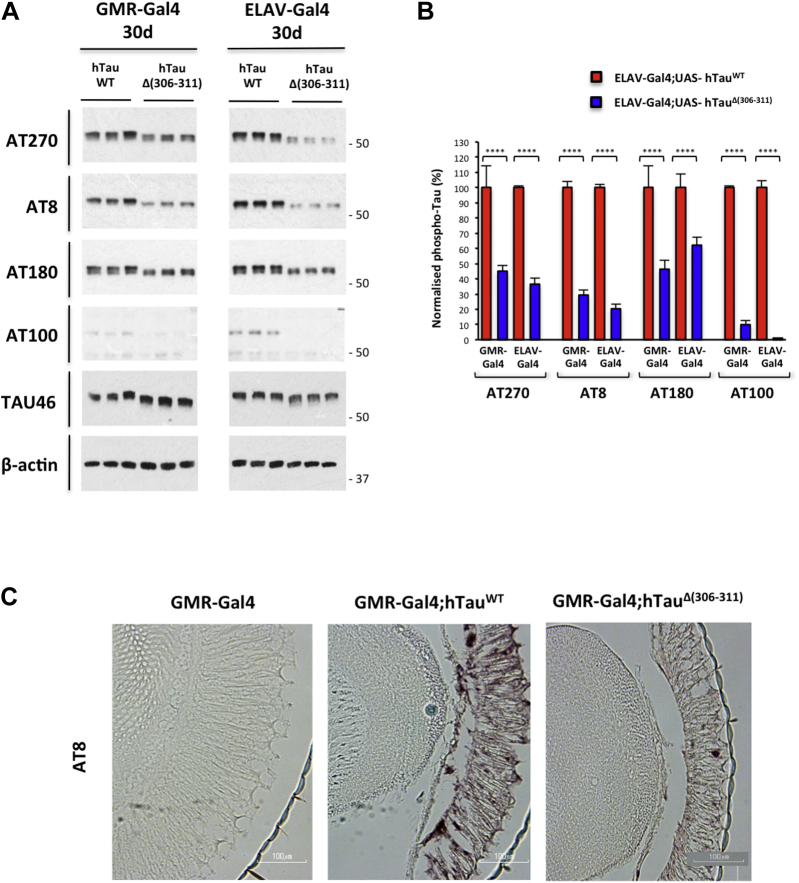
Δ (306–311) in Tau-383 flies reduces Tau phosphorylation. (A) Western blot and (B) quantitation of hTau phosphorylation. The following anti-Tau antibodies were used: Tau46, AT270, AT8, and AT180. Beta-actin served as the control. Two-way ANOVA followed by Turkey's post hoc test: **** p < 0.0001, n = 3 different crosses/genotypes. (C) Immunohistochemistry with AT8 of retina of 30-day-old flies (5 μm paraffin sections). Wild-type (GMR-Gal4) and transgenic (GMR-Gal4;hTau^WT^ and GMR-Gal4;hTau^Δ(306−311)^) flies were analyzed.

Expression of wild-type Tau-383 in retina and brain resulted in marked MC-1 staining (Fig. 2A and B). When Δ306–311 Tau-383 was expressed, staining for MC-1 was markedly reduced (Fig. 2A and B) and resembled that in GMR-Gal4 and ELAV-Gal4 flies. Expression of wild-type Tau-383 in retina and brain caused marked phosphorylation at AT270, AT8, and AT180 epitopes (Fig. 3A and B). When Δ306–311 Tau-383 was expressed in retina and brain, phosphorylation at all 3 epitopes was reduced. No differences between flies expressing wild-type Tau-383 or Δ306–311 Tau-383 were observed with Tau46 (Fig. 3A and B). By immunohistochemistry, a reduction in AT8 staining was present in the eyes of 30-day-old flies with the 306–311 deletion when compared with those expressing wild-type Tau-383 (Fig. 3C).

### 3.3 Deletion of residues 306–311 in Tau-383 flies prevents a rough eye phenotype and reduces brain vacuolization

**Fig. 4:**
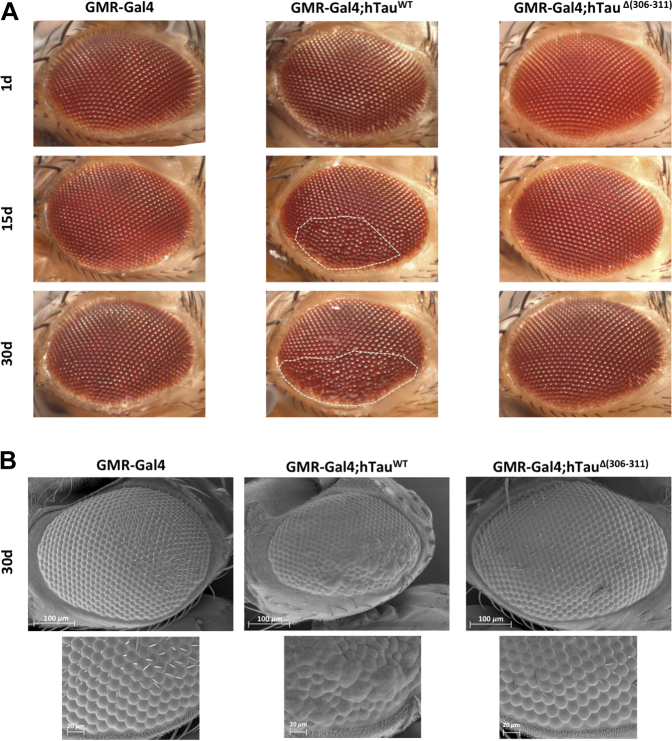
Δ(306–311) in Tau-383 flies abolishes age-related neurodegeneration in the eye. (A) Light microscopy of fly eyes. Unlike Δ306–311 Tau-383 flies, wild-type Tau-383 flies develop a rough eye phenotype before day 15. (B) Scanning electron micrographs of fly eyes. Wild-type Tau-383 flies develop a rough eye phenotype and a reduced eye size, whereas Δ306–311 Tau-383 flies show eyes of normal size with a regular arrangement of ommatidia. A higher magnification of part of the scanning electron micrographs is shown in the bottom panels.

**Fig. 5:**
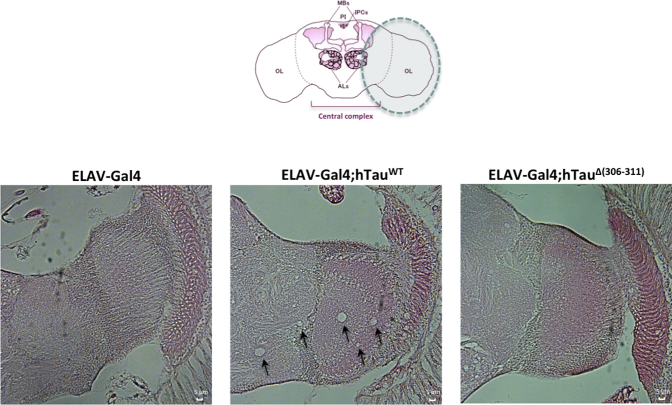
Δ(306–311) in Tau-383 flies abolishes vacuolar pathology in the brain. Hematoxylin/eosin staining shows many vacuoles in ELAV-Gal4;hTau^WT^ flies, with none in ELAV-Gal4;hTau^Δ(306−311)^ flies (5 μm paraffin sections). Vacuolization was absent in ELAV-Gal4 fly brains. Representative images are shown. Scale bar: 5 μm.

Expression of wild-type Tau-383 in the retina using the GMR-Gal4 driver line caused a “rough eye” phenotype characterized by a reduced external crystalline lattice with fused and disordered ommatidia, as well as abnormal mechanosensory bristles (Fig. 4A). The eyes of GMR-Gal4 flies served as controls. The abnormal eye phenotype appeared before 15 days of age, but was not present at day 1 (Fig. 4A). By contrast, expression of Δ306–311 Tau-383 in the retina using the GMR-Gal4 driver line resulted in the formation of normal-looking eyes in flies that were 1, 15, or 30 days old (Fig. 4A). These light microscopic findings were confirmed by scanning electron microscopy of the eyes of 30-day-old flies (Fig. 4B). Expression of wild-type Tau-383 in the brain using the ELAV-Gal4 driver line caused extensive vacuolization (Fig. 5). The brains of ELAV-Gal4 flies served as controls. Thirty-day-old flies were analyzed using hematoxylin/eosin staining of frontal paraffin brain sections. Vacuolization was absent in the brains of age-matched flies expressing Δ306–311 Tau-383 (Fig. 5).

### 3.4 Deletion of residues 306–311 in Tau-383 flies restores climbing activity and normal lifespan

**Fig. 6:**
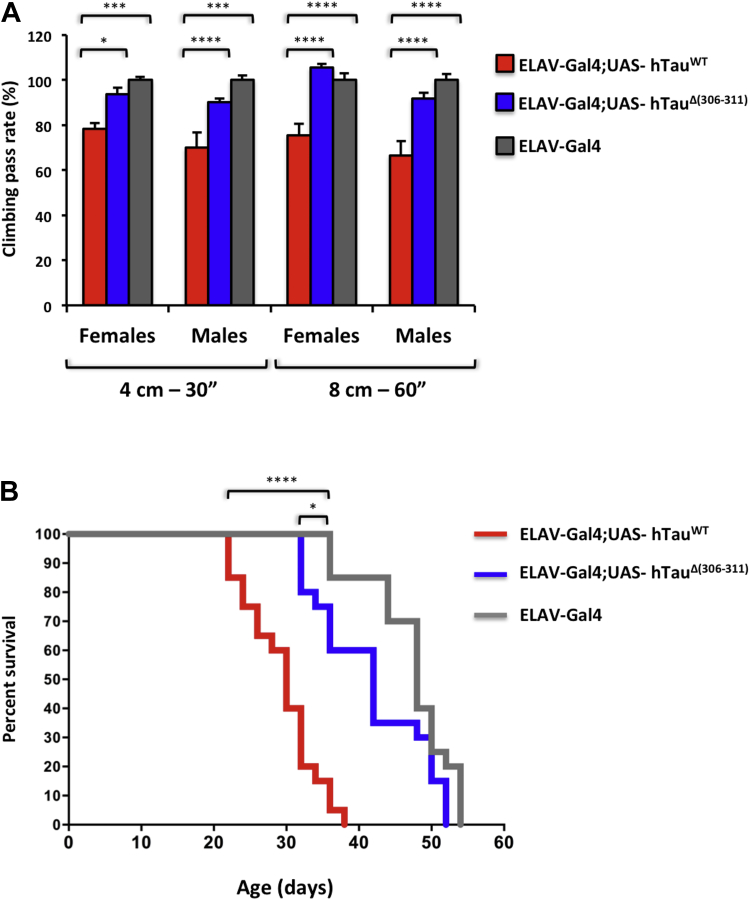
Δ(306–311) in Tau-383 flies normalizes climbing activity and lifespan. (A) Normal climbing activity of ELAV-Gal4;hTau^Δ(306−311)^ flies. The number of flies crossing the 4 cm mark in 30 seconds (left) and the 8 cm mark in 60 seconds (right) was counted. Average climbing activity in 30-day-old ELAV-Gal4;hTau^Δ(306−311)^ flies was not significantly different from that of ELAV-Gal4 flies. ELAV-Gal4;hTau^WT^ flies had a reduced climbing activity. Two-way ANOVA, followed by post hoc test: *p < 0.05; ***p < 0.0005; ****p < 0.0001 (n = 3 different crosses/genotypes). (B) Close to normal lifespan of ELAV-Gal4;hTau^Δ(306−311)^ flies. ELAV-Gal4;hTau^WT^ flies had a reduced lifespan when compared with ELAV-Gal4;hTau^Δ(306−311)^ transgenic flies (35 days vs. 52 days maximum lifespan). The survival times of ELAV-Gal4;hTau^Δ(306−311)^ flies were slightly different from those of ELAV-Gal4 flies. Log-rank (Mantel-Cox) test, ELAV-Gal4;hTau^WT^ versus ELAV-Gal4, ****p < 0.0001. ELAV-Gal4;hTau^Δ306−311^versus ELAV-Gal4, *p = 0.028.

Expression of wild-type Tau-383 in the fly brain using the ELAV-Gal4 driver line impaired mobility in 30-day-old flies, when compared with the ELAV-Gal4 line (Fig. 6A). Both male and female flies were severely impaired in climbing 4 cm in 30 seconds or 8 cm in 1 minute. Deletion of residues 306–311 significantly ameliorated the climbing deficits caused by expression of Tau-383 (Fig. 6A). Expression of wild-type Tau-383 in the brain using the ELAV-Gal4 driver line shortened the lifespan of adult flies (Fig. 6B). The maximum lifespan was around 35 days, as opposed to 54 days for ELAV-Gal4 flies. By contrast, expression of Δ306–311 Tau-383 in brain resulted in a maximum lifespan of around 52 days (Fig. 6B).

### 3.5 Assembly of recombinant Tau-383

After a lag phase of approximately 18 hours, wild-type Tau-383 incubated with heparin gave rise to a sigmoidally shaped curve, based on Thioflavin T fluorescence (Supplementary Fig. 1). By contrast, under the same conditions, Δ306–311 Tau-383 did not give rise to an increase in fluorescence.

## 4 Discussion

Human Tau-induced nerve cell dysfunction and degeneration were previously studied in *Drosophila*. Models were created by expression of human wild-type Tau or Tau with frontotemporal dementia and Parkinsonism linked to chromosome 17 mutations (Ali et al., 2012, Shulman and Feany, 2003, Wittmann et al., 2001). Expression of human Tau in the eye induced a phenotype of small eye, a reduction in retinal thickness and the loss of regular ommatidial organization. Moreover, progressive age-dependent neurodegeneration characterized by nuclear fragmentation and vacuole formation were present in brain. Tau filaments were not observed, indicating that large, insoluble tau aggregates were not required for neurotoxicity.

Residues 306–311 of tau (VQIVYK) are present in the structured cores of tau filaments from AD and Pick's disease brains (Falcon et al., 2018, Fitzpatrick et al., 2017). In recombinantly expressed human Tau, this sequence is necessary for the formation of cross-β-sheets (Li and Lee, 2006, von Bergen et al., 2000). Accordingly, we found that when Δ306–311 0N4R Tau was incubated with heparin, Thioflavin T fluorescence did not change.

To investigate the importance of Tau assembly into β-sheets for neurodegeneration, we produced transgenic flies expressing similar levels of wild-type Tau-383 and Tau-383 lacking residues 306–311 in eye and brain. The line expressing wild-type human Tau exhibited a neurodegenerative phenotype characteristic of other fly models of tauopathy (Ali et al., 2012, Shulman and Feany, 2003, Wittmann et al., 2001). Beginning at about 1 week of age and progressing over time, the eyes developed a rough phenotype characterized by shrinkage with fused and disordered ommatidia, as well as irregular bristles. When Tau was expressed in brain, extensive vacuolization was in evidence. As reported before (Wittmann et al., 2001), we could not detect sarkosyl-insoluble Tau. It follows that β-sheet-rich Tau assemblies caused neurodegeneration, in the absence of large Tau filaments. This effect contrasted markedly with the absence of a rough eye phenotype and vacuolization in brain, when Tau-383 lacking residues 306–311 was expressed. Recombinant Tau with that deletion was unable to assemble into β-sheets.

Retina and brain of wild-type Tau-383 flies were immunopositive for MC-1. By contrast, flies expressing Δ306–311 Tau-383 showed a marked reduction in MC-1 staining. The specificity of MC-1 for pathological Tau is due to a unique conformation, which requires 2 discontinuous intramolecular epitopes that are separated by almost 300 amino acids (^7^EFE^9^ in the N-terminus and ^313^VDLSKVTSKC^322^ in R3) (Carmel et al., 1996, Jicha et al., 1997). MC-1 staining is one of the earliest markers of Tau aggregation (Weaver et al., 2000). NMR experiments using heparin-induced filaments of 4R Tau also showed an interaction between the N-terminus and residues 313–322 of the structured core (Bibow et al., 2011). Moreover, the cryogenic electron microscopy structures of Tau filaments from AD brain showed a density consistent with ^7^EFE^9^ contacting K317 and K321 in the protofilament core (Fitzpatrick et al., 2017). The present findings show that MC-1 reactivity is strongly dependent on the ability of Tau to assemble into β-sheets.

Wild-type Tau-383 was phosphorylated, as evidenced by reactivity with phosphorylation-dependent anti-Tau antibodies AT270, AT8, and AT180. Phosphorylation of these epitopes was reduced in flies expressing Δ306–311 Tau-383. These findings suggest that much Tau phosphorylation was the consequence of the ability of Tau-383 to assemble. They are reminiscent of a study in mice expressing either wild-type human Tau or Tau with mutations I277P and I308P (Lathuiliere et al., 2017). Phosphorylation of Tau may play a part in neurodegeneration because preventing phosphorylation at S/T-P sites has been shown to reduce Tau toxicity in *Drosophila* (Steinhilb et al., 2007).

Locomotor activity was assessed using a climbing assay commonly used for studying the neurotoxic effects of aggregation-prone proteins (Feany and Bender, 2000, Moloney et al., 2010). Flies expressing wild-type human Tau-383 showed a significant reduction in climbing behavior when compared with controls. By contrast, the climbing behavior of flies expressing Δ306–311 Tau-383 was similar to that of controls.

Longevity measurements provide a surrogate for neurological function (Moloney et al., 2010). In flies expressing wild-type Tau-383, there was a significant reduction in lifespan compared with those expressing Δ306–311 Tau-383. There was no significant difference in lifespan between wild-type flies and those transgenic for Δ306–311 Tau-383.

In conclusion, we show that the ability of Tau to assemble into β-sheets is necessary for toxicity. These findings are in agreement with a study showing that chloride-naphthoquinone-tryptophan alleviated toxicity of Tau (Frenkel-Pinter et al., 2017). However, in addition to inhibiting the assembly of ^306^VQIVYK^311^, this compound also reduced the levels of expressed human Tau by 70%. We confirm that Tau-induced neurodegeneration in *Drosophila* does not require the formation of large filaments. Smaller Tau assemblies may be responsible for neurodegeneration. These findings are reminiscent of models of β-amyloidosis (Crowther et al., 2005, Luheshi et al., 2007) and synucleinopathy (Periquet et al., 2007), where protein aggregation was also required for neurodegeneration.

## Disclosure statement

Authors have no conflict of interest to declare.

## Appendix A Supplementary data

Supplementary Fig. 1 β-Sheet assembly of wild-type and Δ306–311 Tau-383. Assembly was monitored using Thioflavin T (ThT) fluorescence for 5 days (120 h). A representative experiment is shown. Similar findings were obtained using 3 different batches of purified recombinant Tau
