# Supplementary figures and images for: Beta-sheet assembly of Tau and neurodegeneration in Drosophila melanogaster
Source: Neurobiol Aging. 2018 Dec;72:98–105. doi: 10.1016/j.neurobiolaging.2018.07.022 (PMC6327151; doi:10.1016/j.neurobiolaging.2018.07.022)

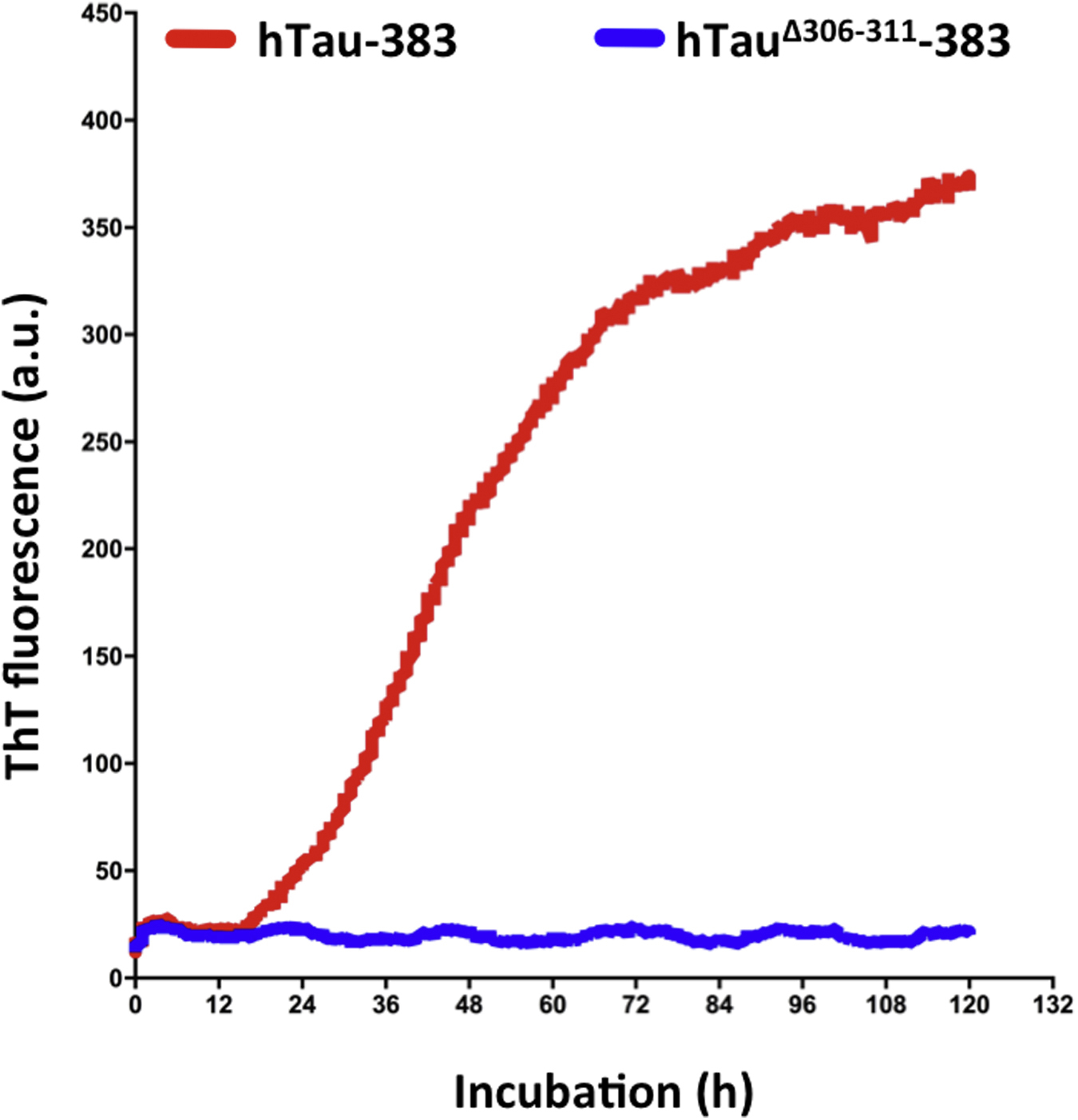

Supplement: Supplementary Fig. 1 — β-Sheet assembly of wild-type and Δ306–311 Tau-383. Assembly was monitored using Thioflavin T (ThT) fluorescence for 5 days (120 h). A representative experiment is shown. Similar findings were obtained using 3 different batches of purified recombinant Tau [file figs1.jpg]
